# Oroxylin A Suppresses the Cell Proliferation, Migration, and EMT via NF-*κ*B Signaling Pathway in Human Breast Cancer Cells

**DOI:** 10.1155/2019/9241769

**Published:** 2019-06-23

**Authors:** XiaoHu Sun, Xinzhong Chang, Yunhua Wang, Boyang Xu, Xuchen Cao

**Affiliations:** ^1^Tianjin Medical University Cancer Institute and Hospital, National Clinical Research Center for Cancer, Tianjin, China; ^2^Key Laboratory of Cancer Prevention and Therapy, Tianjin, China; ^3^Key Laboratory of Breast Cancer Prevention and Therapy, Tianjin Medical University, Ministry of Education, Tianjin, China; ^4^Tianjin University of Traditional Chinese Medicine, China

## Abstract

Oroxylin A is a natural extract and has been reported to have a remarkable anticancer function. However, the mechanism of its anticancer activity remains not quite clear. In this study, we examined the inhibiting effects of Oroxylin A on breast cancer cell proliferation, migration, and epithelial-mesenchymal transition (EMT) and its possible molecular mechanism. The cytoactive and inflammatory factors were analyzed via Cell Counting Kit-8 assay and ELISA assay, respectively. Flow cytometry and western blotting were used to assess the cell proliferation. In addition, a wound healing assay and transwell assay were used to detect cell invasion and migration. qRT-PCR and western blot were employed to determine the effect of Oroxylin A on the EMT formation. Moreover, expression level of protein related to NF-*κ*B signaling pathway was determined by western blot. The results revealed that Oroxylin A attenuated the cytoactivity of MDA-MB-231 cells in a dose- and a time-dependent manner. Moreover, cell proliferation, invasion, and migration of breast cancer cells were inhibited by Oroxylin A compared to the control. The mRNA and protein expression levels of E-cadherin were remarkably increased while N-cadherin and Vimentin remarkably decreased. Besides, Oroxylin A suppressed the expression of inflammatory factors and NF-*κ*B activation. Furthermore, we also found that supplement of TNF-*α* reversed the effects of Oroxylin A on the cell proliferation, invasion, migration, and EMT in breast cancer cells. Taken together, our results suggested that Oroxylin A inhibited the cell proliferation, invasion, migration, and EMT through inactivating NF-*κ*B signaling pathway in human breast cancer cells. These findings strongly suggest that Oroxylin A could be a therapeutic potential candidate for the treatment of breast cancer.

## 1. Introduction

Breast cancer is one of the most common cancers and the second leading cause of cancer death in women. Even if advances in inchoate diagnosis and treatment of breast cancer have been made, approximately 30% of patients with breast cancer will develop cureless metastatic disease[1, 2] in which the median survival times range from 1 to 4 years [3]. Triple negative (TN) breast cancer is a particular type of breast cancer, which lacks the expression of oestrogen receptor (ER), progesterone receptor (PgR), and ERBB2. The features contribute to biological aggressiveness, worse prognosis, and lack of a therapeutic target compared to other subtypes [4]. Thus, triple negative breast cancer is a huge challenge for modern medicine.

The relationship between cancer and inflammation has been studied for many years. Inflammation in the tumor microenvironment plays an extremely important role in tumor progress [5]. In cancer-related inflammation, major inflammatory cytokines (such as IL-1*β*, IL-6, and TNF-*α*) and transcription factors (such as NF-*κ*B and STAT3) are recognized as key endogenous factors [6]. It has been proved that NF-*κ*B participates in initiation and progression of tumor in tissues where cancer-related inflammation easily occurs [7]. NF-*κ*B is not only a key coordinator of inflammation and innate immunity, but also regarded as an important endogenous tumor promoter. In tumor cells, NF-*κ*B induces the expression of antiapoptotic genes to accelerate cell growth and activates relative inflammatory cytokines, adhesion molecules, and angiogenic factors to expedite the proliferation of tumor cells [8–11].

Oroxylin A (OA) is natural compound extracted from* Scutellariae radix*. It has been proved that the compound exerts broad functions, including anticancer, anti-inflammation, neuroprotective, and anticoagulation [12]. The functions have attracted much attention wisely, especially the antitumor activity. OA performs a strong anticancer activity by inducing apoptosis, inhibiting metastasis and invasion, reversing multidrug resistance, suppressing angiogenesis, and so on [13]. In a previous study, OA could suppress the expression of Bcl-2 and procaspase-3 protein in HepG2 cell significantly to exert proapoptotic effect [14]. OA suppressed cell invasion and had antimetastatic effect through downregulating the expression of MMP2 and MMP9 in MDA-MB-435 human breast cancer cells [15]. Moreover, OA prevented inflammation-related tumor through downregulation of inflammatory gene expression by inhibiting NF-*κ*B signaling pathway [5]. Further, OA inhibited activated transcription factor NF-*κ*B, reducing the expression of LPS-induced iNOS and COX-2 in RAW 264.7 cells [16]. A large number of researches about the anticancer effects of OA have been studied. However, it is unclear whether and how OA can exert the anticancer effects on TN breast cancer by NF-*κ*B signaling pathway.

Thus, we explored the inhibiting effects of OA on the proliferation, migration, and EMT on MDA-MB-231 human breast cancer cells in this study and reported the protective effects of OA via suppressing NF-*κ*B signaling pathway.

## 2. Materials and Methods

### 2.1. Reagents and Antibodies

Oroxylin A was bought from Novartis Pharmaceuticals (Basel, Switzerland) and dissolved in dimethyl sulfoxide (DMSO) as a stock solution. Primary antibodies against CDK2, CyclinE, p27, p-p65, p65, and COX-2 were obtained from Cell Signaling (Beverly, MA, USA). Primary antibodies against E-cad, N-cad, Vimentin, p-I*κ*B, I*κ*B, and GAPDH were obtained from Cell Signaling Technology (Danvers, MA). Secondary antibodies conjugated with horseradish peroxidase were obtained from Santa Cruz Biotechnology. All other reagents were purchased from Sigma-Aldrich (Louis, MO, USA).

### 2.2. Cell Culture

The human breast cancer cells MDA-MB-231 were obtained from the American Type Culture Collection (ATCC, Manassas, VA) and maintained in Dulbecco's modified Eagle's medium (DMEM; Gibco, Gaithersburg), supplemented with 10% fetal bovine serum, penicillin 100U/ml, streptomycin 100 *μ*g/ml, and incubated in a humidified incubator at 37°C and 5% CO_2_. Cells were divided into four groups: the control (treated with PBS), TNF*α* (treated with TNF-*α*), OA20 (treated with 20*μ*M Oroxylin A), and OA20+TNF*α* (treated with 20*μ*M Oroxylin A+TNF-*α*).

### 2.3. Cell Viability Assay

The effect of OA on MDA-MB-231 cell growth was measured by CCK-8 assay. Cells were seeded into 96-well plates and treated with OA at various concentrations (0*μ*M, 10 *μ*M, 20 *μ*M, and 40 *μ*M). Each concentration was repeated five times. After 24 and 48 hours, 10 *μ*l CCK-8 (Dojindo, Kumamoto, Japan) reagent was added to each well. Plates were then estimated on a microplate reader after 2 h incubation. Absorbance was measured at 450 nm using microplate spectrophotometry.

### 2.4. Flow Cytometric Analysis

Flow cytometry was used to explore the cell proliferation. Treated with OA (20 *μ*M) or/and TNF-*α* for 24 h, the cells were harvested and centrifuged at 300 g for 5 min. Then, the cells were stained with a Cell Cycle Detection Kit (KeyGene, Holland) according to the manufacturer's instructions. Finally, the samples were analyzed with the FACSCalibur Flow Cytometer (BD Bioscience, USA) and BD CellQuest software (BD Bioscience, USA).

### 2.5. Western Blotting

MDA-MB-231 cells were treated with OA (20 *μ*M) or/and TNF-*α* for 24h, harvested, and homogenized in 200 *μ*L RIPA lysis buffer. The concentration of protein was determined by the protein quantitative kit. Then, the proteins samples were separated by in SDS-PAGE and transferred to PVDF membrane. After blocked with 5% of skimmed milk powder for 2 h, the PVDF membranes were incubated with the primary antibodies (CDK2, 1:1000, #2546; CyclinE, 1:1000, #4129; p27, 1:1000, #3688; E-cadherin, 1:1000, #14472; N-cadherin, 1:1000, #13116; Vimentin, 1:1000, #5741; p-p65, 1:1000, #3036; p65, 1:1000, #8242; p-I*κ*B, 1:1000, #2859; I*κ*B, 1:1000, #4814; COX-2, 1:1000, #12282; GAPDH, 1:1000, #5174) overnight at 4°C. Then, the membranes were cultured with secondary horseradish peroxidase-labeled antibody at 37°C for 1 h. The target proteins on the membranes were analyzed using ECL Blotting Detection Reagents.

### 2.6. Wound Healing Assay

Cell migration capacity was calculated by a wound healing assay. Cells were seeded in 6-well plates and allowed to growth to 80–90% confluence. The cell monolayers were then wounded with white pipette tips and washed four times with phosphate-buffered saline to remove cell debris. The cells were then incubated in medium with OA (20 *μ*M) or/and TNF-*α* for 24 h. Cell migration into the wound surface and number of migrated cells were determined under an inverted microscopy. Five randomly chosen fields were analyzed in each well.

### 2.7. Cell Invasion Assay

An invasion assay was implemented to examine tumor invasion using transwell chamber (6.5 mm in diameter, 8 *μ*m pore-size, Corning Costar, Cambridge, MA). Firstly, the transwell chambers were first loaded with 0.1mL of matrigel (Becton Dickinson, Bedford, MA) at 37°C for 1 h. MDA-MB-231 cells were incubated in DMEM containing 1% FBS and treated with OA (20 *μ*M) or/and TNF-*α* for 24h. Then cells were trypsinized and suspended at a final concentration of 5×10^5^ cells/mL in DMEM containing 1% FBS. Cell suspensions were then loaded into the upper compartment, and medium with 10% fetal bovine serum was added in the lower compartment. Incubated at 37°C in 5% CO_2_ for 24 h, cells on the upper surface were wiped off with a cotton swab. Then, invaded cells on the lower surface were fixed, stained, and counted under a microscope. Five randomly chosen fields were counted for each group.

### 2.8. Quantitative Real-Time PCR

Cells were pretreated with 20*μ*M OA for 24 h. Total RNA was extracted by TRIzol (Invitrogen, Carlsbad, CA) reagent. The purity and quality of RNA were examined by NanoDrop2000 (Thermo Scientific, Wilmington, DE). Total RNA was reversely transcribed into cDNA using the Reverse Transcription System Kit (Takara; Dalian, China). Then Quantitative real-time PCR was carried out using SYBR green qPCR kit (TakaraBio, Inc.) on an ABI PRISM 7900 Real-Time system (Applied Biosystems, Foster City, CA, USA). The PCR amplification program was the following: 95°C for 20 sec, followed by 40 cycles of 95°C for 1 sec and 60°C for 20 sec. The sequences of PCR primers were listed as follows: E-cadherin (forward: 5′-CCACCAAAGTCACGCTGAAT-3′, reverse: 5′-GGAGTTGGGAAATGTGAGC-3′), N-cadherin (forward: 5′-GTGCCATTAGCCAAGGAATTCAGC-3′, reverse: 5′-GCGTTCCTGTTCCACTCATAGGAGG-3′), Vimentin (forward: 5′-ATGAAGGTGCTGCAAAAC-3′, reverse: 5′-GTGACTGCACCTGTCTCCGGTA-3′), GAPDH (forward: 5′-ATG AGCCCCAGCCTTCTCCAT-3′, reverse: 5′-GGTCGGAGTCAACGGATTTG-3′). mRNA level was expressed as the relative change after normalized versus GAPDH. Relative expression was quantified by the 2^−ΔΔCq^ method [17]. The expression levels were relative to the fold change of the controls, which were defined as 1.0.

### 2.9. ELISA Assay

IL-6, IL-8, and TNF-*α* secretion in cell supernatants was measured by ELISA according to the manufacturer's instructions. 6 replicates were established for each group and results were from triplicate experiments.

### 2.10. Statistical Analysis

SPSS 20.0 statistical analysis software was used to analyze the experimental data. The results were expressed as mean ± SD. Statistical comparisons were made by two-tailed Student's t-test or one-way analysis of variance (ANOVA). P < 0.05 was considered statistically significant.

## 3. Results

### 3.1. Effect of OA on Cell Viability of Breast Cancer Cells

**Figure 1:**
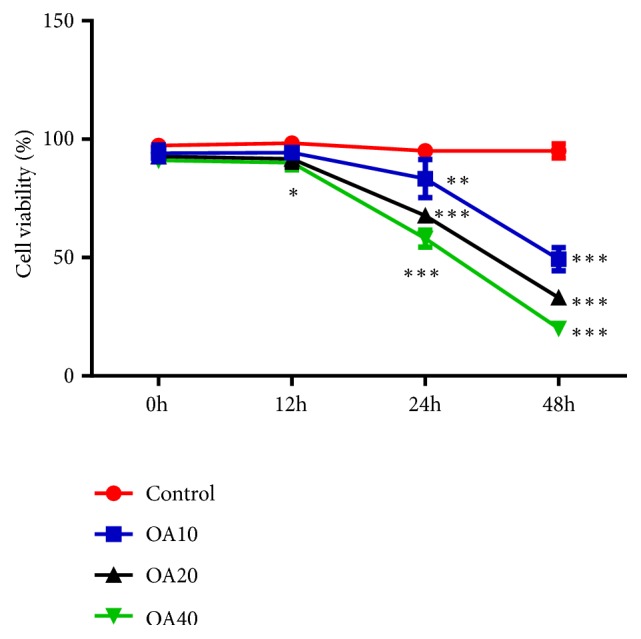
Effect of Oroxylin A on growth of MDA-MB-231 cells at different time points. Data are expressed as mean ± SD.^*∗*^P < 0.05, ^*∗∗*^P < 0.01, ^*∗∗∗*^P < 0.001 versus control.

To investigate the effect of OA on the growth of MDA-MB-231 cells, CCK-8 assay was performed. The results showed that OA inhibited the growth of MDA-MB-231 cells in a dose- and a time-dependent manner (Figure 1). Based on the above-mentioned result, a 24-hour treatment of OA at the concentration of 20 *μ*M was applied to the subsequent experiments.

### 3.2. Effect of OA on the Proliferation of Breast Cancer Cells

**Figure 2:**
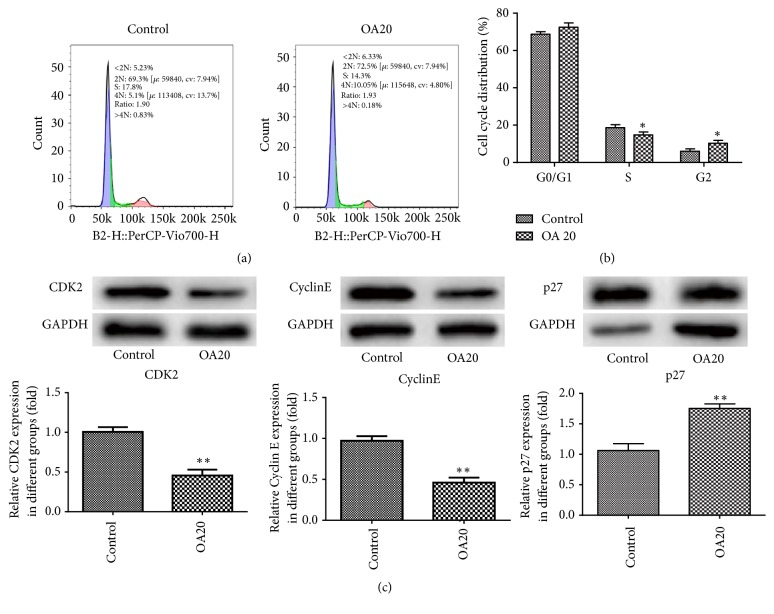
Effect of Oroxylin A on proliferation of MDA-MB-231 cells. (a-b) Flow cytometry assays were performed to analyze the cell cycle progression when MDA-MB-231 cells were treated with OA 24 h later. (c) The expressions of proliferation-related protein CDK2, CyclinE, and p27 were detected by western blot analysis. Data are expressed as mean ± SD. ^*∗*^P< 0.05, ^*∗∗*^P < 0.01 versus control.

To determine whether OA would influence the proliferation of MDA-MB-231 cells, flow cytometry and western blotting were used. As shown in Figures 2(a) and 2(b), OA had obvious effects on the cell cycle. OA treatment increased the G1 phase population while decreasing the S phase population in MDA-MB-231 cells. Furthermore, the results of western blot indicated that the protein expression of CDK2 and Cyclin E was extremely decreased compared to the control, while p27 had the opposite results (Figure 2(c)).

### 3.3. Effect of OA on the Migration and Invasion of Breast Cancer Cells

**Figure 3:**
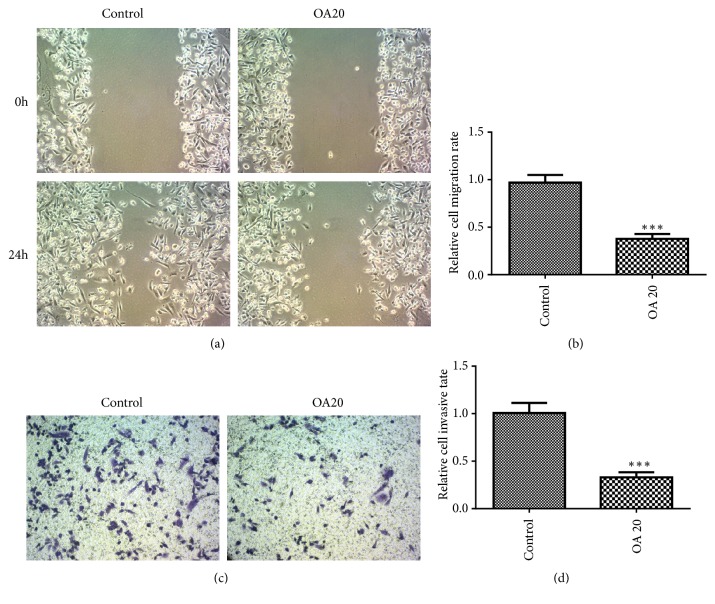
Effect of Oroxylin A on MDA-MB-435 cell migration and invasion in vitro. (a) Oroxylin A inhibits the cell migration. The cells were seeded in a 6-well plate and cell monolayer was wounded and washed by PBS and then incubated with 20 *μ*M Oroxylin A for 24 h. Migration was assessed by microscope with a camera. Image magnification: 100×. (c) Oroxylin A inhibits the cell invasion. Cells were cultured in the presence of 20 *μ*M Oroxylin A for 24 h and seeded in the upside of transwell with matrigel for 24 h. The lower side of the membrane was stained and counted under a microscope. Image magnification: 100×. Data are expressed as mean ± SD. ^*∗∗∗*^P < 0.001 versus control.

Wound healing assay and transwell assay were applied for investigating the effect of OA on the migration and invasion of MDA-MB-231 cells, respectively. The results showed that cells in the control rapidly spread to the wound area after 24 h incubation, whereas very few cells in OA group spread forward (Figure 3(a)). Additionally, the results from invasion assay revealed that numerous cells invaded through the matrigel in the control, while the number of cells invading to the lower side significantly decreased in the treatment of OA (Figure 3(c)). And the relative cell invasive and migration rates in OA group were both dramatically lower than the control (Figures 3(b) and 3(d)).

### 3.4. Effect of OA on EMT Formation

**Figure 4:**
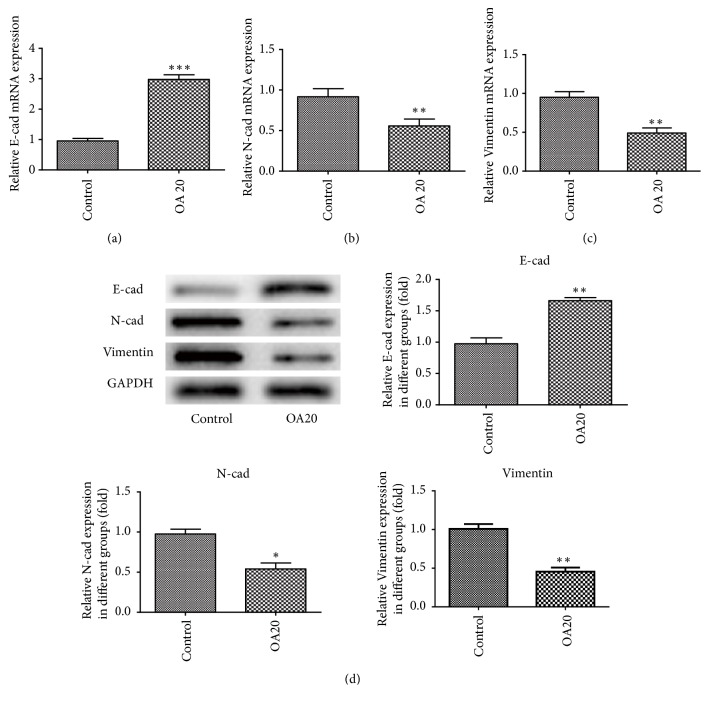
Effect of Oroxylin A on EMT formation in MDA-MB-231 cells. Cells were treated with 20 *μ*M Oroxylin A for 24 h. E-cadherin (a), N-cadherin (b), and Vimentin (c) mRNAs were measured with qRT-PCR. (d) The expression of E-cadherin, N-cadherin, and Vimentin proteins in the cells was analyzed by western blotting using specific antibodies. Data are expressed as mean ± SD. ^*∗*^P < 0.05, ^*∗∗*^P < 0.01, ^*∗∗∗*^P < 0.001 versus control.

To examine the effect of OA on the formation of EMT, the expression of E-cadherin, N-cadherin, and Vimentin in MDA-MB-231cells was detected by qRT-PCR and western blotting analysis. It was shown that the mRNA and protein expression levels of E-cadherin remarkably elevated, whereas the expression of N-cadherin and Vimentin was considerably decreased when compared to the control (Figure 4). These results suggest that EMT formation could be suppressed by OA.

### 3.5. Effect of OA on the Expression of Inflammatory Cytokines

**Figure 5:**
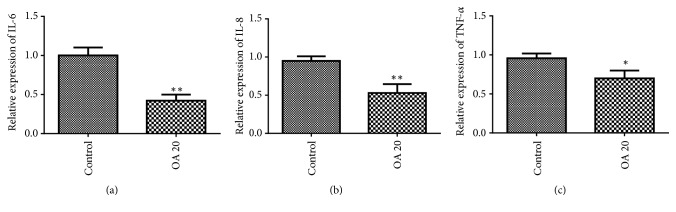
Effect of Oroxylin A on IL-6, IL-8 and TNF-*α* levels in MDA-MB-231 cells. The expression of IL-6, IL-8, and TNF-*α* was detected by ELISA assay from the supernatants of cells cultured with 20 *μ*M Oroxylin A for 24 h. Data are expressed as mean ± SD. ^*∗*^P < 0.05, ^*∗∗*^P < 0.01 versus control.

To explore whether inflammatory cytokines were inhibited in breast cancer in response to OA, the levels of IL-6, IL-8, and TNF- *α* were detected by ELISA. The results showed that OA markedly suppressed the protein expressions of IL-6, IL-8, and TNF-*α* in MDA-MB-231cells, compared with the control (Figure 5).

### 3.6. Inhibitory Effect of OA on NF-*κ*B Signaling Pathway

**Figure 6:**
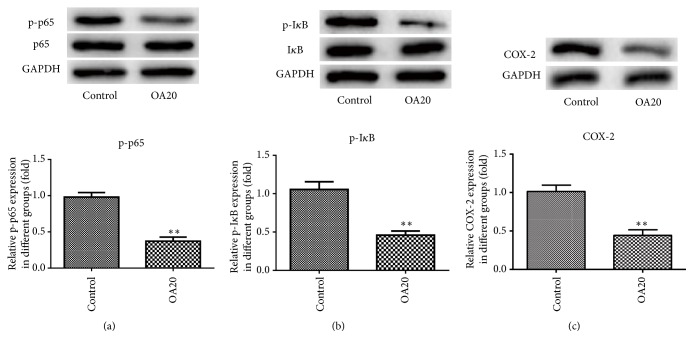
Effect of Oroxylin A on NF-*κ*B activation and the expression of COX-2 in MDA-MB-231 cells. Phosphorylation of p65 (a) and I*κ*B (b) and COX-2 (c) expression were analyzed by western blot. GAPDH was used as an internal control. Data are expressed as mean ± SD. ^*∗∗*^P < 0.01 versus control.

NF-*κ*B signaling pathway has been recognized to play vital roles in the regulating of inflammatory pathways in the tumor microenvironment. Thus, the effects of OA on NF-*κ*B activity were assessed by western blotting. As shown in Figure 6, OA did not affect the total amounts of p65 and I*κ*B but significantly inhibited the phosphorylation of them. Furthermore, the protein level of COX-2 was markedly decreased after treatment with OA compared to the control. These results indicated that OA effectively attenuated activation of the NF-*κ*B pathway with the repression of COX-2 in breast cancer cells.

### 3.7. TNF-*α* Reverses the Functions of OA on TN Breast Cancer Cells

**Figure 7:**
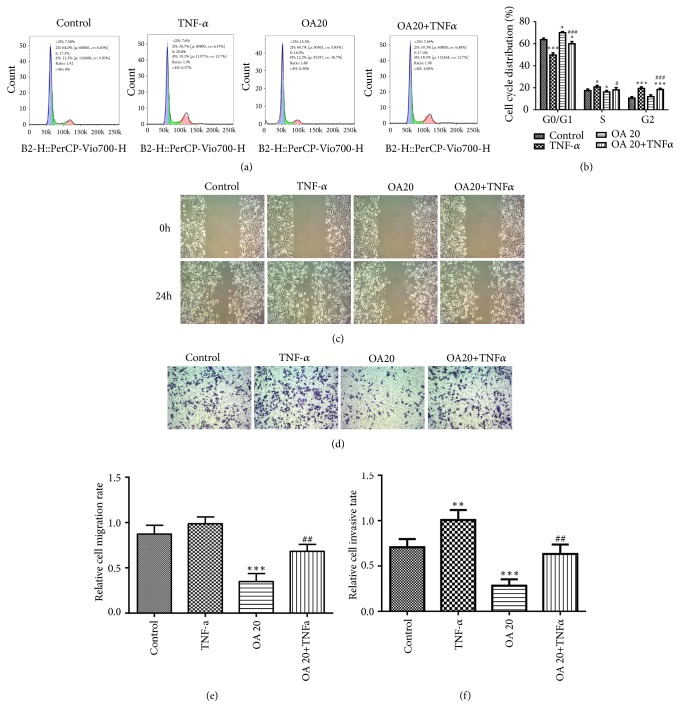
TNF-*α* reverses the function of OA in breast cancer cells. (a-b) Flow cytometry, (c) wound healing assay, and (d) transwell assay were performed in MDA-MB-231 cells treated with control, TNF-*α*, OA, and OA + TNF*α*. Image magnification: 100×. Data are expressed as mean ± SD. ^*∗*^P < 0.05, ^*∗∗*^P < 0.01, ^*∗∗∗*^P < 0.001 versus control; ^#^P < 0.05, ^##^P < 0.01, ^###^P < 0.001 versus the OA control.

**Figure 8:**
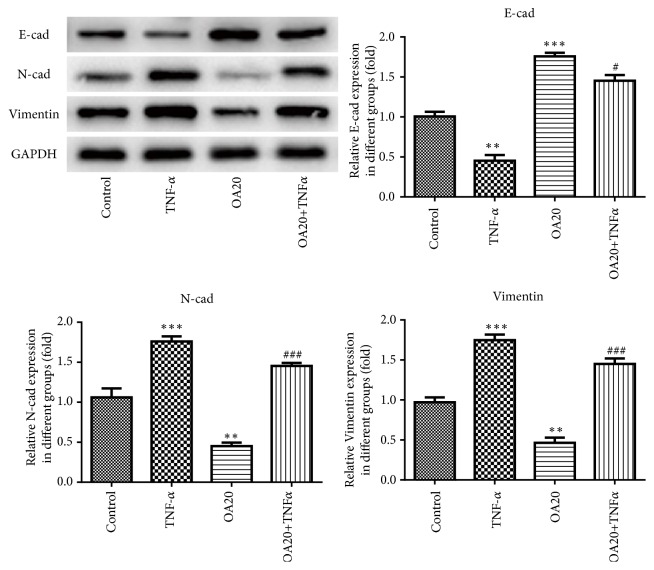
TNF-*α* reverses the effect of Oroxylin A on EMT formation in MDA-MB-231 cells. The protein expressions of E-cadherin, N-cadherin, and Vimentin in the cells were analyzed by western blotting. Data are expressed as mean ± SD. ^*∗∗*^P < 0.01, ^*∗∗∗*^P < 0.001 versus control; ^#^P < 0.05, ^###^P < 0.001 versus the OA control.

TNF stimulates the growth of normal mammary epithelial cells and the mammary cancer cells [18]; thus we studied the effects of TNF-*α* on the proliferation, migration, and EMT of breast cancer cells with or without OA. Flow cytometry demonstrated that OA suppressed and TNF-*α* promoted cell proliferation compared to the control, while the OA +TNF*α* group showed that OA-inhibited cell proliferation was promoted by TNF-*α* (Figures 7(a) and 7(b)). In addition, the results from wound healing assay and transwell assay showed that OA suppressed and TNF-*α* promoted cell migration and invasion, while treatment of OA +TNF*α* promoted OA-inhibited cell migration and invasion (Figures 7(c) and 7(d)) than the OA group. Furthermore, the results from a western blot revealed that OA inhibited and TNF-*α* enhance EMT phenotype formation of MDA-MB-231 cells while the OA +TNF*α* group reversed the EMT formation inhibited by OA (Figure 8). Taken together, these findings suggest that the effects of OA on the suppression of proliferation, migration, and EMT formation on breast cancer cells could be reversed by TNF-*α*.

## 4. Discussion

OA, one of the main bioactive flavonoids of* Scutellariae radix*, exerts obvious antitumor activities [19]. It has been proved that OA inhibited breast cancer progression by proapoptosis, antiproliferation, antiangiogenesis, and so on [15, 20–22]. However, the mechanism of OA defenses against breast cancer remains not to be fully elucidated.

In the present study, we found that OA significantly reduced the cytoactivity of MDA-MB-231 cells in a dose-dependent manner. Moreover, compared with the control, OA inhibited proliferation, migration, and EMT formation and the expression of inflammatory cytokines of breast cancer cells obviously. The experiments also showed that OA inhibited NF-*κ*B activation by suppressing the phosphorylation of p65 and I*κ*B, as well as the expression of COX-2. Furthermore, it was found that TNF-*α* reversed the effects of OA on breast cancer cells.

The inflammatory antitumor response plays a critical role in inhibiting tumor growth in human cancers [23]. NF-*κ*B is one of the most important regulators of proinflammatory gene expression [24], contributing to the progress of tumor. Recent studies have revealed that NF-*κ*B is involved in regulation of expression of COX-2 [25]. Cyclooxygenase-2 (COX-2), a rate-limiting enzyme, regulates the formation of carcinogens, apoptosis, and angiogenesis in tumor progression [26]. Previous study showed that Oroxylin A modulated NF-*κ*B signaling to hold back inflammation related tumor [5]. Moreover, it is reported that Oroxylin A suppressed LPS-induced expression of iNOS and COX-2 related to inflammatory processes by inhibition of NF-*κ*B activation in macrophages [27]. In the present study, we found that OA significantly suppressed the activation of NF-*κ*B signaling in MDA-MB-231cells by suppressing phosphorylation of I*κ*B and p-65, which are important steps in NF-*κ*B activation. What is more, the protein expression of COX-2 was found to be decreased, which may be associated with inhibition of NF-*κ*B signaling.

Chronic inflammation is a main activator in the metastatic cascade [28]. Epithelial-mesenchymal transition (EMT) is a complex process, losing cell-cell adhesion, contributing to cancer invasion and metastasis. The results of the present study indicated that OA inhibited the EMT formation in breast cancer cells by upregulation of E-cadherin levels and downregulation of N-cadherin and Vimentin levels. EMT programs stimulate the production of proinflammatory factors in cancer environment while inflammation is a potent inducement for EMT in tumors [9]. What is more, NF-*κ*B activity promotes the collection of inflammatory cells and secretion of proinflammatory cytokines like IL-1, IL-6, IL-8, and TNF-*α* [29]. The present results demonstrated that OA markedly suppressed the secretion of IL-6, IL-8, and TNF-*α* protein in MDA-MB-231cells. These findings indicate OA has a repressive influence on EMT and inflammation progress in breast cancer cells.

TNF-*α* has been proved to have pleiotropic effects on cells death, growth, or differentiation under different concentrations and context [30, 31]. TNF has anticancer and anti-infection effects in high concentrations because of its cytotoxic effects on the tumor vessel [32]. However, it is shown that TNF can promote the proliferation and migration of normal epithelial cell and breast cancer cells both at low doses [33, 34]. One study demonstrated that TNF-*α* secreted by tumor cells stimulated the growth of malignant mammary epithelial cells, which contributed to mammary tumorigenesis in neu/erbB2 transgenic mice [18]. Another study showed that TNF-*α* can promote transendothelial migration of breast cancer via upregulation of LOX [35]. In the present study, we found OA-suppressed cell proliferation, migration, invasion, and EMT phenotype formation were promoted by TNF-*α*, leading to aggravation of breast cancer process, which is consistent with previous reports. On these bases, we hypothesized that the blockade of TNF-*α* may enhance the antitumor effects of OA for breast cancer patients and this speculation warrants further investigation.

## 5. Conclusion

In summary, the present study showed that OA could suppress cell proliferation, migration, invasion, and EMT formation and downregulate the expressions of inflammatory cytokines. Furthermore, OA exerted protective effect on breast cancer by inhibiting NF-*κ*B signaling pathway. The results provided a theoretical foundation for Oroxylin A as a potential drug for the treatment of breast cancer.

## Data Availability

The data used to support the findings of this study are included within the article.
